# Mapping the expansion of coyotes (*Canis
latrans*) across North and Central America

**DOI:** 10.3897/zookeys.759.15149

**Published:** 2018-05-22

**Authors:** James W. Hody, Roland Kays

**Affiliations:** 1 North Carolina State University, Department of Forestry and Environmental Resources, 2800 Faucette Drive, Raleigh, NC, USA 27607; 2 North Carolina Museum of Natural Sciences, Nature Research Center, 9 West Jones Street, Raleigh, NC, USA 27601

**Keywords:** coyote, *Canis
latrans*, range expansion, museum records, FAUNMAP, VertNet, historical ecology, Holocene

## Abstract

The geographic distribution of coyotes (*Canis
latrans*) has dramatically expanded since 1900, spreading across much of North America in a period when most other mammal species have been declining. Although this considerable expansion has been well documented at the state/provincial scale, continent-wide descriptions of coyote spread have portrayed conflicting distributions for coyotes prior to the 1900s, with popularly referenced anecdotal accounts showing them restricted to the great plains, and more obscure, but data-rich accounts suggesting they ranged across the arid west. To provide a scientifically credible map of the coyote’s historical range (10,000–300 BP) and describe their range expansion from 1900 to 2016, we synthesized archaeological and fossil records, museum specimens, peer-reviewed reports, and records from wildlife management agencies. Museum specimens confirm that coyotes have been present in the arid west and California throughout the Holocene, well before European colonization. Their range in the late 1800s was undistinguishable from earlier periods, and matched the distribution of non-forest habitat in the region. Coyote expansion began around 1900 as they moved north into taiga forests, east into deciduous forests, west into costal temperate rain forests, and south into tropical rainforests. Forest fragmentation and the extirpation of larger predators probably enabled these expansions. In addition, hybridization with wolves (*C.
lupus*, *C.
lycaon*, and/or *C.
rufus*) and/or domestic dogs has been documented in the east, and suspected in the south. Our detailed account of the original range of coyotes and their subsequent expansion provides the core description of a large scale ecological experiment that can help us better understand the predator-prey interactions, as well as evolution through hybridization.

## Introduction

During the past century, coyotes have undergone a dramatic range expansion across much of North and Central America. Previously restricted to the western two-thirds of North America, the species now occurs across most of the continent, from the Atlantic to the Pacific seaboard and from Alaska to Panama (Macdonald and Sillero-Zubiri 2004). Despite widespread management as a pest species (Andelt 1987, Knowlton et al. 1999), coyotes have nevertheless expanded their geographic range by an estimated 40% since the 1950s, at least twice as much any other North American carnivore during the same time period (Laliberte and Ripple 2004).

Various interacting factors are thought to have contributed to coyotes’ rapid expansion in North America. First, extirpation of apex predators likely helped coyotes expand by reducing predation risk and allowing coyotes to expand their niche to larger prey. Specifically, the extirpation of wolves (*C.
lupus*, *C.
rufus*, and/or *C.
lycaon*) and cougar (*Puma concolor*) across most of eastern North America, and the decline of cougar and jaguar (*Panthera
onca*) in Central America probably set the stage for coyote colonization (Bekoff and Gese 2003, Berger and Gese 2007, Cove et al. 2012, Méndez-Carvajal and Moreno 2014). Second, conversion of once-forested landscapes to agricultural landscapes in eastern North America and Central America likely facilitated coyote expansion by creating suitable coyote habitat in areas that were previously unsuitable (Vaughan 1983, Parker 1995, Macdonald and Sillero-Zubiri 2004). The expansion of coyotes into western Canada and Alaska has been attributed to the creation of new human settlements during gold rushes in the late 1880s (Gier 1975, Moore and Parker 1992), although this explanation has not been critically tested. Additionally, hybridization of coyotes with wolves and domestic dogs in eastern North America introduced new genotypes that may have promoted colonization and survival in eastern habitats (Kays et al. 2010, VonHoldt et al. 2011, Thornton and Murray 2014). Coyotes expanding into the southeastern United States likewise bear evidence of introgression from dogs (Adams et al. 2003). There is currently no evidence of coyote hybridization with dogs or wolves in the northwestern flank of their expansion, but coyotes moving into Central America are suspected to be hybridizing with dogs based on morphological characters (Cove et al. 2012, Hody 2016).

This ongoing range expansion poses an excellent case study in community ecology and acclimation or adaptation in the Anthropocene, and also presents a new challenge for conservation, as the ecological implications of spreading coyotes are still largely unknown. Coyotes may represent a new top predator in eastern North America and other parts of the continent, with cascading effects on predator communities and disease dynamics (Gompper 2002, Levi et al. 2012). Likewise, the recent arrival of coyotes in Panama may position them to colonize South America, with unknown implications for tropical ecosystems (Hidalgo-Mihart et al. 2004, Méndez-Carvajal and Moreno 2014, Hody 2016). Rigorously testing the causes and consequences of coyote range expansion requires an accurate historical context for where the species previously occurred. However, current accounts of coyote distribution suffer from two major problems.

First, the historic distribution of coyotes prior to the westward expansion of European settlers in the 1800s has recently been confused in the literature. This confusion is largely due to misinterpretation of a figure from Moore and Parker (1992) and Parker (1995). In these publications, the authors provide a general depiction of historical coyote ranges before and after European colonization of North America. In contrast to the authors’ detailed written descriptions of subsequent coyote range expansion in eastern North America, these continent-wide maps were conceptual illustrations of an existing historical narrative and did not assess actual coyote occurrence data. More accurate coyote range maps have been published in the past (e.g., Young and Jackson 1951, Nowak 1978, 1979), but the Parker (1995) map has recently been reproduced as accurate description of coyote range expansion in the scientific and popular literature (e.g. Levy 2012).

**Figure 1. F1:**
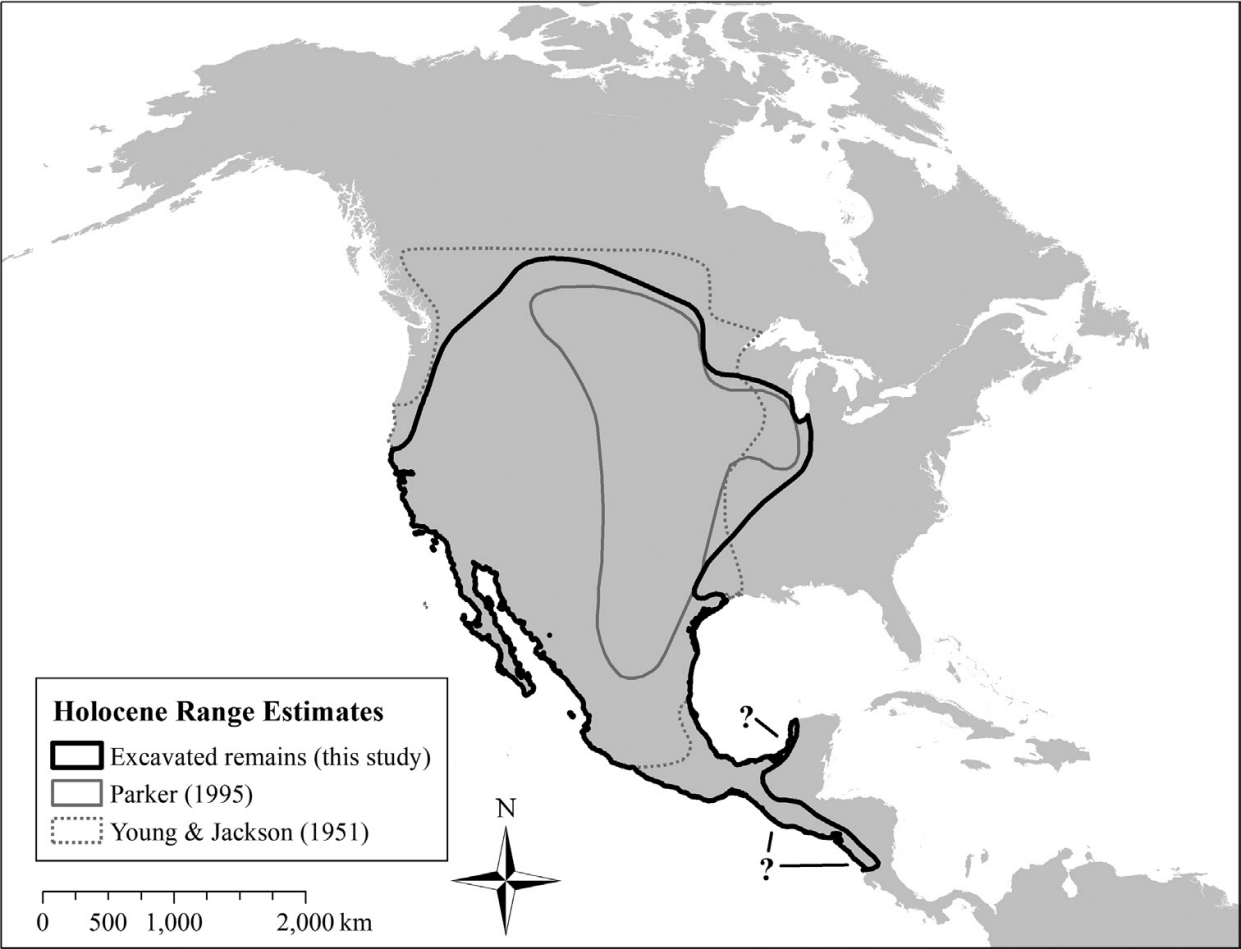
Comparison of Holocene coyote range maps, pre-expansion. Fossil and zooarchaeological remains suggest that coyotes were distributed throughout western North America prior to European colonization, contrary to widely-cited accounts (e.g., Parker 1995).

In this popular narrative, coyotes were restricted to true prairie ecosystems prior to European settlement (Figure 1), bounded between the Mississippi River and the Rocky Mountains from southern Canada to central Mexico (Moore and Parker 1992, Parker 1995). The extirpation of wolves and land conversion by Europeans then presumably allowed a westward expansion of coyotes in the late 1800s, followed by a series of eastern expansions during the 1900s (Moore and Parker 1992, Parker 1995, Levy 2012).

However, range maps based on physical evidence (Nowak 1978, 1979), historical accounts, and coyote specimens in California suggests a wider western distribution. Grinnell et al. (1937) indicated that coyotes occurred in California well before European settlement, with the exception of a few heavily forested counties along the northern California seacoast, which coyotes colonized during the early 1900s. Numerous accounts by Native Americans and early European colonists confirm the presence of coyotes in California, as do zooarchaeological remains (e.g., Young and Jackson 1951 and references therein). Moreover, the genetic structure of Californian coyote populations suggest that they occurred in the area well before European colonization (Sacks et al. 2004), contradicting the hypothesis of a recent westward expansion.

Additionally, the original northern and southern range limits of coyotes remain uncertain in both narratives (Nowak 1978, Moore and Parker 1992). In Alaska and northern Canada, authors have debated whether coyotes historically occurred in low densities, arrived during the 1880s, or arrived during the 1900s (Nowak 1978, Parker 1995, MacDonald and Cook 2009). The original southern extent of coyote range has been similarly controversial. Fossil evidence confirms that coyotes were present in the Yucatán Peninsula and northwestern Costa Rica during the Pleistocene (Lucas et al. 1997, Arroyo-Cabrales and Alvarez 2003), but their southern distribution after late-Pleistocene climatic changes is less clear. In their seminal work on coyote ecology, Young and Jackson (1951) suggested that coyotes only recently colonized Central America, although the written accounts of coyote-like canids in the 1500s and late 1800s provide anecdotal evidence otherwise (Monge-Nájera and Morera Brenes 1987, Hidalgo-Mihart et al. 2004). Pre-Columbian coyote remains have also been found in at least two sites in the Yucatán Peninsula, lending credibility to this hypothesis (Hidalgo-Mihart et al. 2004). Overall, the historical distribution of coyotes during the Holocene remains poorly characterized and warrants reexamination.

A second problem with existing large-scale accounts of coyote range is that the recent expansion of coyotes has been coarsely described, without clear spatiotemporal detail. Maps are typically offered without citing reference material, and with few, widely scattered time intervals. Consolidating and improving continent-wide descriptions of coyote range expansion would facilitate testing hypotheses about their effects on newly colonized ecosystems.

Fortunately, coyotes are well represented in museum collections, having been hunted extensively due to their abundance and widespread reputation as a nuisance species. Furthermore, coyotes are also well represented in the fossil and zooarchaeological record, allowing inferences about their distribution several thousand years ago. We compiled museum records from recent biological surveys, fossil and zooarchaeological collections, peer-reviewed literature, and management agency reports to characterize the historical distribution of coyotes prior to European settlement and catalogue their expansion decade-by-decade from 1900 to 2016.

## Materials and methods

We compiled coyote occurrences from two data repositories: VertNet (Constable et al. 2010) and the Quaternary Faunal Mapping Project, FAUNMAP (Graham and Lundelius 2010). These repositories allow ecological inferences at two different time-scales. FAUNMAP documents fossil and zooarchaeological coyote remains (hereafter, “excavated remains”) throughout the Quaternary period, providing occurrence records across deep time scales. Conversely, VertNet documents coyote specimens collected during biological surveys of live animals (e.g., skins, skeletons, taxidermy animals, tissue samples; hereafter, “preserved specimens”) and allows inferences about the distribution of coyotes from the mid-1800s through the present. Both data sources provide spatially and temporally referenced coyote occurrences across North America, collectively documenting their distribution over the past 10,000 years.

For our query in FAUNMAP, we searched for excavated remains of coyotes (*Canis
latrans*) from the Holocene epoch (10,000-0 years before present, BP). Taxonomically modern coyotes (*C.
latrans*) also occurred in the late Pleistocene, but biomes and faunal assemblages present in North America at the time drastically differed from those of the Holocene (Van Valkenburgh and Hertel 1993, Williams et al. 2004), with measurable effects on the ecological niche of the coyote itself (Meachen and Samuels 2012, Meachen et al. 2014, Pardi and Smith 2016). We therefore focus on their Holocene distribution, considering their Pleistocene range a separate but closely related topic.

Our query in VertNet considered preserved specimens of coyotes (*Canis
latrans*), coydogs (C.
latrans
×
familiaris), and coywolves (C.
latrans
×
lycaon/*lupus*/*rufus*) that were collected during 1850–2016. We restricted our query to records that included information about the year and location where the specimen was collected. For quality control reasons, we only considered specimens that included georeferenced point coordinates or enough locality information to reference the data to a specific county. Coyote records from Mexico collected between 1850–1899 were retained as an exception to this rule, because more precise data were not available. In these cases, we allowed records that were referenced to at least a state-level.

In addition to these specimen records, we also compiled first-occurrence records and fossil records of coyotes from peer-reviewed literature and reports by state wildlife management agencies (references listed in Suppl. material 1). For first-occurrence records, we favored observations that were associated with either physical specimens (e.g. from hunters and trappers) or archived photographs (e.g., from camera traps) wherever possible, although we also considered other reputable first-hand accounts in areas where data were sparse. These records proved particularly valuable in defining the expansion of coyotes in Central America and the southeastern United States. For fossil and zooarchaeological records, we searched peer-reviewed reports of excavated coyote remains from Mexico and Central America, dated to 10,000–300 years BP. These records supplement FAUNMAP, the spatial coverage of which is limited to the United States and Canada. Since fewer records of excavated remains are available for this region, it is more difficult to clearly define the southernmost historical limit of coyotes. However, these records provide some indication of the Holocene distribution of coyotes in Central America. Other types of data (e.g., Native American folklore, narrative accounts of European settlers) might further elucidate the historical range of coyotes. However, we restricted our inferences in this study to physical specimens, scientific literature, and management agency records, which can be more readily referenced to a specific spatial location and time interval. All the raw coyote occurrence data are available through Data Dryad (http://doi: 10.5061/dryad.1qp358p).

We used these datasets to create two maps. First, we sought to clarify the Holocene distribution of coyotes before large-scale settlement by Europeans using FAUNMAP and a subset of the VertNet data (collected 1850–1899). We also identify which FAUNMAP records had a known minimum age >300 BP to permit stronger inference. Second, we used data from VertNet, peer-reviewed literature, and state management agencies to develop a highly detailed map of 20^th^ century coyote range expansion at 10-year intervals. In both cases, we approximated range boundaries for each historical period (Holocene, 1900, 1910, etc.) by manually constructing polygons around occurrence records from the corresponding time interval.

During the 20^th^ century, coyotes were occasionally brought into areas by hunters and trappers prior to natural expansion into the area (Parker 1995). These introductions produced isolated coyote records ahead of the colonizing front, but coyote populations in these areas usually did not persist (Fener et al. 2005, Kays et al. 2010). To avoid including these populations in our analysis, we excluded extreme spatial outliers from our distribution map (e.g., an isolated record might be omitted if it occurred in an area with known historical introductions and no neighboring records occurred within 500 km for many years).

In the Holocene figure, we also displayed coarse approximations of potential forest cover based on Ramankutty and Foley (2010). We included this layer to visually illustrate the spatial distribution of historical coyote specimens in relation to dominant land cover types. We defined potential forest cover as areas where tropical, temperate, or boreal forests would have occurred in the area based on large-scale estimates by Ramankutty and Foley (2010). We caution that the historical extent of forest cover in North and Central America contracted and expanded considerably prior to European contact due to the agriculture activities, settlement building, and land burning practices of Pre-Columbian civilizations (Denevan 1992, Kimmerer and Lake 2001, Cook et al. 2012). Thus, potential forest cover should not be interpreted as a literal, static depiction of American land cover throughout the Holocene. Instead, it should be interpreted as a general index for areas where forest cover frequently or intermittently occurred over several thousand years.

## Results

**Figure 2. F2:**
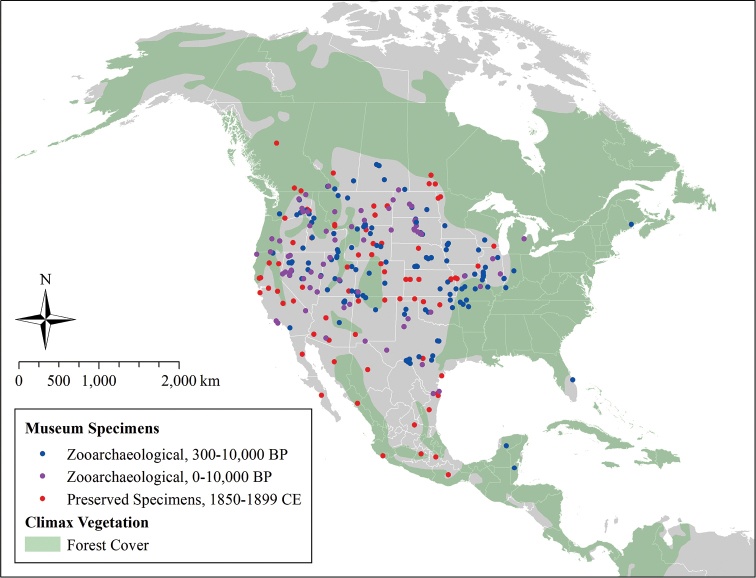
Historical distribution of coyotes from 10,000 years before present (BP) to 1899. Zooarchaeological (FAUNMAP) records document the distribution of coyotes during the Holocene (0–10,000 BP).

Our query in FAUNMAP yielded 347 records from the United States and Canada with specific data on the minimum and maximum age of the coyote remains. These were distributed between the Pacific Ocean and the Mississippi River, with the exception of two spatial outliers occurring in New Brunswick, Canada and Florida, USA (Figure 2). It is possible that these two records reflect a more widespread eastern distribution of coyotes in the Holocene. However, we find it more likely that they reflect misidentified remains of related *Canis* sp.

Our query in VertNet yielded 12,319 records of coyotes and coyote hybrids from North and Central America, providing specimen-vouchered coyote occurrences from 1850-2016. Among these records, 4,949 were already georeferenced, and an additional 3,523 records had sufficient locality information to reference the data to individual counties or corresponding political units. An additional 3,747 records could only be referenced to the state- or province-level. We retained such occurrence records for Mexico to address the dearth of available data prior to 1900, but omitted these records elsewhere due to the availability of higher-quality county-level data. Only 100 records had no useable locality information.

### Holocene distribution (10,000 BP–1899)

The spatial distribution of coyote specimens from the late 1800s was similar to the distribution of coyote remains older than 300 BP. Specifically, coyotes extended east to Mississippi and Ohio Rivers and west through California and the arid west (Figure 2). These data indicate that that coyotes’ range in the late-1800s reflected a longstanding geographic distribution that formed well before the 1700s, not a recent westward expansion. This contradicts widely-cited descriptions of the historical distribution of coyotes (Figure 1), which suggest that California and the Rocky Mountains as areas that were colonized by coyotes as recently as the 19^th^ and 20^th^ centuries (Moore and Parker 1992, Parker 1995, Levy 2012). Instead, the historical distribution of coyotes matches areas where non-forested habitats (e.g., grassland, prairie, desert) dominate the climax vegetation, more closely corresponding to earlier range descriptions by Nowak (1978, 1979, 2002) and Young and Jackson (1951). The Holocene distribution of coyotes in Mesoamerica remains unclear due to the relatively small number of published historical specimens available from this area.

### Contemporary expansion (1900–2016)

**Figure 3. F3:**
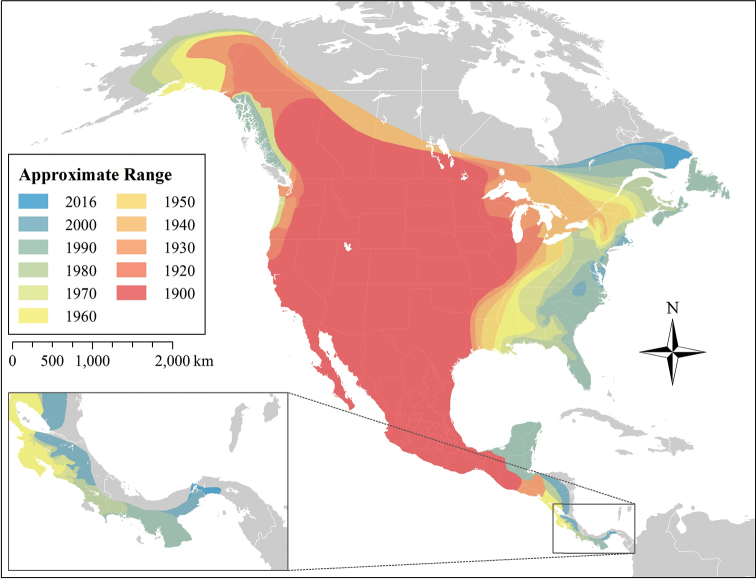
Coyote range expansion by decade, 1900–2016. Ranges are based on occurrence of museum specimens, peer-reviewed literature with associated specimens or photographs, and reports from state wildlife management agencies. The distribution of coyotes between the Yucatán Peninsula and Nicaragua is coarsely depicted due to the paucity of available data, representing the earliest confirmed occurrence. All referenced materials are listed in Suppl. material 1.

Combining museum records and regional coyote literature, we created a detailed continent-wide description of coyote range expansion at 10 year intervals (Figure 3). This map consolidates previous efforts and corrects popular misconceptions about the magnitude of coyotes’ expansion in the west. Additionally, it provides the first account of coyote range expansion at this level of spatial and temporal detail. We offer this as a starting point for future discussions and encourage further improvements to this map wherever local data might become available. Additional research is needed in some areas, particularly Central America and the Mid-Atlantic United States, where historical records are sparse.

## Discussion

We compiled coyote occurrences from past biological surveys, fossils, zooarchaeological records, and existing literature to document the historical distribution of coyotes throughout the Holocene and reconstruct decade-by-decade range expansion during 1900–2016. Our findings indicate that coyotes historically occupied a larger area of North America than generally suggested in recent literature, more closely matching the historical range presented by Young and Jackson (1951) and Nowak (1978, 1979) than Parker (1995) (Figure 1). Our results closely resemble the written range description by Nowak (1979), which assesses coyotes as having “a wide distribution, primarily in the western half of the continent” prior to European contact, with unknown range limits but extending “at least as far east as southern Wisconsin, northwestern Indiana, western Arkansas, and central Texas.”

The distribution of excavated coyote remains 10,000–300 BP matches the distribution of preserved coyote specimens collected between 1850 and 1899 almost identically, suggesting that the geographic range of coyotes in the late 1800s had already been established prior to the 1700s. This same spatial pattern emerged when FAUNMAP data were subdivided in other ways, suggesting that this was not an artifact of how we defined our time intervals. Importantly, Holocene coyote remains ≥4,000 BP showed the same general pattern presented in Figure 2, confirming the presence of coyotes as far east as Arkansas and central Texas, as far south as the Yucatán Peninsula, and as far west as California. These records predate the rise of North and Central American civilizations with large permanent settlements (e.g., Olmec, Aztec, Mayan, Mississippian) (Kuiper 2010), suggesting that coyotes were widely distributed throughout the Holocene independent of large-scale land use change by Pre-Columbian civilizations.

Excavated coyote remains and 19^th^ century museum records occurred throughout most non-forested habitats in North America. These specimen records show that coyotes occurred in the Rocky Mountains and Arid West throughout the Holocene, contradicting the proposed western expansion of coyotes during the late-1800s (Parker 1995), although there was a smaller expansion into forests of the Pacific Northwest in the early 1900s.

The distribution of excavated remains includes four notable outliers, warranting further discussion: one in southern Florida, one in New Brunswick, and two on the Yucatán Peninsula. Although we consider the New Brunswick sample questionable, the Florida and Yucatán specimens might reflect historical range dynamics of coyotes. The Florida record is dated to the early Holocene, but its estimated range age overlaps with the late Pleistocene as well. Coyote fossils from this geological epoch have been documented across the Florida peninsula (Graham and Lundelius 2010), which was previously dominated by grassland ecosystems (Feranec and MacFadden 2000, Feranec 2004). This record likely reflects coyote occurrence in the late Pleistocene, or misidentified red wolf remains from the early Holocene. Alternatively, it might indicate that coyotes briefly persisted in the savannah habitats of southern Florida after forest habitats arose elsewhere in eastern North America. The New Brunswick record is much younger, referring to mandibles found in a Native American shell midden from the year 830 ± 65 BP. While this is possible that these remains represent an extreme eastern distribution of coyotes in the past (Stewart 1976), we suspect that they may be misidentified remains from domestic dogs, which were also found on site and appear in similar deposits from New England (Ingraham 2011).

The two Yucatán specimens, both noted by Hidalgo-Mihart et al. (2004), suggest a historical presence of coyotes in parts of Central America, and possible range expansion associated with Mayan land use and deforestation. The westerly record is dated to the early Holocene (Arroyo-Cabrales and Alvarez 2003), suggesting a longstanding presence of coyotes in the area (Hidalgo-Mihart et al. 2004). This record occurs near relatively open habitat along the western coast of the Yucatán Peninsula (Ramankutty and Foley 2010), possibly facilitating their historical presence there. The eastern record is much younger, associated with Postclassic Mayan ruins in Belize (Emery 1999), and may indicate that coyotes existed in areas deforested by the Maya civilization (Hidalgo-Mihart et al. 2004). Interestingly, written accounts noted by Monge-Nájera and Morera Brenes (1987) and Hidalgo-Mihart et al. (2004) spatially coincide with areas that most heavily cultivated and deforested prior to European contact (Cook et al. 2012).

We cannot definitively assess the Holocene southern limit of coyotes due to paucity of data in Central America. However, we generally agree with Hidalgo-Mihart et al. (2004) that coyotes may have existed in naturally occurring open habitats and Pre-Columbian agricultural areas of Central America prior to the 1500s based on available records, contrasting earlier descriptions (Young and Jackson 1951). We hypothesize that the southern distribution of coyotes might have fluctuated during the Holocene, with populations extending eastward across the Yucatán Peninsula and southward along the Pacific coast of Central America in periods when barriers of forested habitat were broken, either naturally or by agricultural activities of Mesoamerican civilizations. Additional research is needed to clarify their historical distribution of coyotes south of Mexico, but all available evidence suggests that this species was restricted to habitats north of the Nicoya Peninsula in northwestern Costa Rica until the mid-1900s (Vaughan 1983).

Our map of coyote records from 1900-2016 shows how and when coyotes expanded their range into forested biomes. Agriculture was widespread in these previously forested regions by 1900, so this more open, fragmented landscape presumably aided their expansion, although Kays et al. (2008) note that eastern coyotes now occur in large forested wilderness, and thus are not reliant on open habitats. Our map also reflects the relatively rapid colonization of the northeast in comparison with the southeast, which Kays et al. (2010) suggested was due to higher levels of wolf introgression allowing a more rapid evolution of larger body size. More recently, VonHoldt et al. (2016) showed that wolf genes associated with body size have been positively selected for in eastern coyotes, and rapidly spread throughout the eastern population. Coyotes now occur through eastern North America, and are now expanding to isolated islands with recent sightings in the Florida Keys (Greene and Gore 2013) and Long Island, New York (Weckel et al. 2015).

Although coyote range expansion into eastern Canada has been well studied (Crête and Desrosiers 1995, Crête et al. 2001, Patterson and Messier 2003, Chubbs and Phillips 2005), historical reasons for the northward expansion of coyotes into western Canada and Alaska described in the literature remain sparse. This early northwestern expansion is generally attributed to land clearing and refuse left by settlers during the gold rushes of the late 1800s (Gier 1975, Moore and Parker 1992). This explanation appears chronologically appropriate, but it is doubtful that these disturbances alone would provide coyotes with enough momentum to establish resident populations in western Canada and further colonize southeastern Alaska in the 1900s. Interestingly, coyotes have now established at least one breeding population in the Taiga Shield ecozone, near Yellowknife, Northwest Territories (Cluff 2006). It is unclear whether this population extends into undeveloped areas, or if it is restricted to disturbed habitats (Cluff 2006).

Likewise, coyote expansion southward across Central America is also not well studied. Coyotes rapidly expanded into deforested habitats in eastern Panama (Méndez-Carvajal and Moreno 2014, Hody 2016), and the dense forests of the Darién now represent the last major barrier between coyote populations and South American savannah ecosystems (Hidalgo-Mihart et al. 2004, Méndez-Carvajal and Moreno 2014). However, this barrier may be more permeable than previously thought, especially along the coastlines, raising concerns that coyotes might reach South America in the near future (Hody 2016). If coyotes reach South America, it is likely that the grassland and agricultural habitats in Colombia and Venezuela could support viable populations, unless competition with native carnivores restricts them. Observations in eastern Panama suggests that road construction and agricultural development might facilitate coyote range expansion in previously forested tropical landscapes (Méndez-Carvajal and Moreno 2014, Hody 2016), but we find it improbable that coyotes would expand into intact parts of the Amazon rainforest. Conversely, we speculate that the open habitats of the Andes might offer suitable coyote habitat in such a scenario, and allow further expansion around the Amazon. Regardless of its extent, coyote colonization of South America would be an event of profound ecological significance; barring direct introductions by humans, expansion of a North American predator into South American ecosystems has not been observed since the Great American Biotic Interchange 3 million years ago (Wallace 1876, Simpson 1980, Marshall et al. 1982, Leigh et al. 2014), and its potential effects on native wildlife is entirely unknown.

## Conclusion

The expansion of coyotes across the American continent offers a natural experimental system for assessing ecological questions related to their roles as predators, and evolutionary questions related to their hybridization with dogs and wolves. By collecting and mapping all historical and fossil records of coyotes we were able to correct old misconceptions of their original range, and more precisely map and date their recent expansions. We hope these maps will provide useful context for future research into the ecology and evolution of this incredibly adaptive carnivore.

## References
