# Supplementary material for: Mapping the expansion of coyotes (Canis latrans) across North and Central America
Source: Zookeys. 2018 May 22;(759):81–97. doi: 10.3897/zookeys.759.15149 (PMC5974007; doi:10.3897/zookeys.759.15149)
Supplement: Supplementary material 1 — Detailed list of references and data sources [file zookeys-759-081-s001.docx]

**Appendix A. List of references used to determine historical extent and regional first-occurrences of coyotes (*Canis latrans*) in North and Central America.**

**VertNet References:**

American Museum of Natural History (AMNH) Mammal Collection. American Museum of Natural History. http://ipt.vertnet.org:8080/ipt/resource.do?r=amnh_mammals (accessed on 2016-05-13).

California Academy of Sciences Mammalogy Collection (CAS-MAM). California Academy of Sciences, San Fransisco. http://ipt.calacademy.org:8080/ipt/resource.do?r=mam#rights (accessed on 2016-05-13).

Chicago Academy of Sciences (CHAS) Mammalogy Collection. Chicago Academy of Sciences. http://ipt.vertnet.org:8080/ipt/resource.do?r=chas_mammals (accessed on 2016-05-13).

Cornell University Museum of Vertebrates (CUMV) Mammal Collection. Cornel University Museum of Vertebrates. http://ipt.vertnet.org:8080/ipt/resource.do?r=cumv_mamm (accessed on 2016-05-13).

Field Museum of Natural History (Zoology) Mammal Collection (FMNH-M). Field Museum of Natural History. http://fmipt.fieldmuseum.org:8080/ipt/resource.do?r=fmnh_mammals (accessed on 2016-05-13).

Humboldt State University (HSU) Wildlife Mammals. Humboldt State University. http://ipt.vertnet.org:8080/ipt/resource.do?r=hsu_wildlife_mammals (accessed on 2016-05-13).

Illinois State Museum (ISM) Mammalogy Collection. Illinois State Museum. http://ipt.vertnet.org:8080/ipt/resource.do?r=ism_mammals (accessed on 2016-05-13).

Kansas University (KU) Mammalogy Collection. Kansas University Biodiversity Institute. http://ipt.nhm.ku.edu/ipt/resource.do?r=kubi_mammals (accessed on 2016-05-13).

Michigan State University (MSU) Mammalogy, Ornithology and Vertebrate Paleontology Collections. Michigan State University. http://ipt.vertnet.org:8080/ipt/resource.do?r=msu_mammals_birds_vertpaleo (accessed on 2016-05-13).

Museum of Vertebrate Zoology (MVZ) Mammal Collection, University of California, Berkeley. University of California, Berkeley. http://ipt.vertnet.org:8080/ipt/resource.do?r=mvz_mammal (accessed on 2016-05-13).

Lund Museum of Zoology (MZLU). Museum of Zoology of Lund, Sweden. http://www.gbif.se/ipt/resource.do?r=mzlu (accessed on 2016-05-13).

North Carolina Museum of Natural Sciences (NCSM) Mammals Collection. North Carolina Museum of Natural Sciences. http://ipt.vertnet.org:8080/ipt/resource.do?r=ncsm_mammals (accessed on 2016-05-13).

Natural History Museum (London) Collection Specimens. Natural History Museum (London). http://portal.vertnet.org/p/natural-history-museum (accessed on 2016-05-13).

Sam Noble Oklahoma Museum of Natural History (SNOMNH) Mammals Specimens. Sam Noble Oklahoma Museum of Natural History. http://65.52.215.125/ipt/resource.do?r=mammals (accessed on 2016-05-13).

Puget Sound Museum (PSM) Vertebrates Collection. James R. Slater Museum of Natural History. http://ipt.vertnet.org:8080/ipt/resource.do?r=psm_verts (accessed on 2016-05-13).

Santa Barbara Museum of Natural History (SBMNH) Vertebrate Collection. Santa Barbara Museum of Natural History. http://ipt.vertnet.org:8080/ipt/resource.do?r=sbmnh_verts (accessed on 2016-05-13).

Cowan Tetrapod Collection at the University of British Columbia Beaty Biodiversity Museum (UBCBBM). University of British Columbia Beaty Biodiversity Museum. http://ipt.vertnet.org:8080/ipt/resource.do?r=ubc_bbm_ctc_mammals (accessed on 2016-05-13).

University of Florida (UF) Florida Museum of Natural History. University of Florida. http://ipt.flmnh.ufl.edu:8080/ipt/resource.do?r=mammals (accessed on 2016-05-13).

University Museum of Zoology Cambridge (UMZC) Zoological Specimens. University Museum of Zoology Cambridge. http://ipt.vertnet.org:8080/ipt/resource.do?r=umzc_vertebrates (accessed on 2016-05-13).

**Reports and Journal Articles (North America):**

Bragina E, Kays R, Hody J, Moorman C, DePerno C, Mills L (in review) No region-wide effects on white-tailed deer following eastern coyote colonization.

Chubbs TE, Phillips FR (2005) Evidence of range expansion of eastern coyotes, *Canis latrans*, in Labrador. Canadian Field-Naturalist 119: 381–384. doi: 10.22621/cfn.v119i3.149

Crête M, Desrosiers A (1995) Range expansion of coyotes, *Canis latrans*, threatens a remnant herd of caribou, *Rangifer tarandus*, in southeastern Québec. Canadian Field-Naturalist 109: 227–235.

Fener HM, Ginsberg JR, Sanderson EW, Gompper ME (2005) Chronology of range expansion of the Coyote, *Canis latrans*, in New York. Canadian Field-Naturalist 119: 1–5. doi: 10.22621/cfn.v119i1.74

Georges S (1976) A range extension of the coyote in Quebec. Canadian Field-Naturalist 90: 78-79.

Georgia Department of Natural Resources (1991) Census and surveys of coyote populations in Georgia, Statewide Wildlife Survey, Project No. W-47, Study No. V, 16 pp.

Hill EP, Sumner PW, Wooding JB (1987) Human influences on range expansion of coyotes in the southeast. Wildlife Society Bulletin 15 (4): 521-524.

MacDonald SO, Cook JA (2009) Recent mammals of Alaska. University of Alaska Press, Fairbanks, 387 pp.

New Jersey Upland Wildlife and Furbearer Research Project (2012) Range expansion of the eastern coyote in New Jersey.

Patterson BR, Messier F (2003) Age and condition of deer killed by coyotes in Nova Scotia. Canadian Journal of Zoology 81: 1894–1898. doi: 10.1139/z03-189

Weeks JL, Tori GM, Shieldcastle MC (1990) Coyotes (*Canis latrans*) in Ohio. Ohio Journal of Science 90 (5): 142-145.

**Reports and Journal Articles (Central America):**

Bermúdez SEC, (2013) Ticks (Acari: Ixodidae, Argasidae) of coyotes in Panama. Systematic and Applied Acarology 18 (2): 112-115.

Cove M V, Pardo V LE, Spínola RM, Jackson VL, Sáenz JC (2012) Coyote *Canis latrans* (Carnivora: Canidae) Range extension in northeastern Costa Rica: Possible explanations and consequences. Latin American Journal of Conservation 3: 82–86.

Emery KF (1999) Continuity and variability in Postclassic and colonial animal use at Lamanai and Tipu, Belize. In: White CD (Ed), Reconstructing ancient Maya diet. University of Utah Press, Salt Lake City, 61–82.

Hidalgo-Mihart MG, Contreras-Moreno FM, Pérez-Solano LA, Hernández-Lara C (2013) Primeros registros de coyote (*Canis latrans*) en Campeche, México. Revista Mexicana de Biodiversidad 84: 1012-1017.

Hody JW (2016) Canid collision - Range expansion by coyotes (*Canis latrans*) and crab-eating foxes (*Cerdocyon thous*) in Panama and interpretation of camera trap data. MS thesis, Raleigh, USA: North Carolina State University.

Lucas SG, Alvarado GE, Vega E (1997) The Pleistocene mammals of Costa Rica. Journal of Vertebrate Paleontology 17: 413–427. doi: 10.1080/02724634.1997.10010985

Méndez-Carvajal P, Moreno R (2014) Mammalia, Carnivora, Canidae, *Canis latrans* (Say, 1823): Actual distribution in Panama. Check List 10: 376–379. doi: 10.15560/10.2.376

Ordóñez-Garza N, Bulmer W, Eckerlin RP, Matson JO (2008) Coyotes (*Canis latrans*) in Guatemala. Southwestern Naturalist 53 (4): 507-509.

Peña-Mondragón JL, Castillo Álvarez A, Benítez-Malvido J (2014) Primer registro de coyote (*Canis latrans*) en la región de La Selva Lacandona, Chiapas, México. Acta Zoológica Mexicana 30 (3): 696-700.

Platt SG, Miller BW, Miller CM (1998) First record of the coyote (*Canis latrans*) in Belize. Vida Silvestre Neotropical 7: 139-140.

Sosa-Escalante J, Hernández S, Segovia A, Sánchez-Cordero V (1997) First record of the coyote, *Canis latrans*, in the Yucatan Peninsula, Mexico. Southwestern Naturalist 42 (4): 494-495.

Vaughan C (1983) Coyote range expansion in Costa Rica and Panama. Brenesia 21: 27–32.
